# Accurate Estimation of Fungal Diversity and Abundance through Improved Lineage-Specific Primers Optimized for Illumina Amplicon Sequencing

**DOI:** 10.1128/AEM.02576-16

**Published:** 2016-11-21

**Authors:** D. Lee Taylor, William A. Walters, Niall J. Lennon, James Bochicchio, Andrew Krohn, J. Gregory Caporaso, Taina Pennanen

**Affiliations:** a Department of Biology, University of New Mexico, Albuquerque, New Mexico, USA; b Department of Molecular Biology and Genetics, Cornell University, Ithaca, New York, USA; c Broad Institute of MIT & Harvard, Cambridge, Massachusetts, USA; d Department of Biological Sciences, Northern Arizona University, Flagstaff, Arizona, USA; e Natural Resources Institute Finland (Luke), Vantaa, Finland; USDA Forest Products Laboratory

## Abstract

While high-throughput sequencing methods are revolutionizing fungal ecology, recovering accurate estimates of species richness and abundance has proven elusive. We sought to design internal transcribed spacer (ITS) primers and an Illumina protocol that would maximize coverage of the kingdom Fungi while minimizing nontarget eukaryotes. We inspected alignments of the 5.8S and large subunit (LSU) ribosomal genes and evaluated potential primers using PrimerProspector. We tested the resulting primers using tiered-abundance mock communities and five previously characterized soil samples. We recovered operational taxonomic units (OTUs) belonging to all 8 members in both mock communities, despite DNA abundances spanning 3 orders of magnitude. The expected and observed read counts were strongly correlated (*r* = 0.94 to 0.97). However, several taxa were consistently over- or underrepresented, likely due to variation in rRNA gene copy numbers. The Illumina data resulted in clustering of soil samples identical to that obtained with Sanger sequence clone library data using different primers. Furthermore, the two methods produced distance matrices with a Mantel correlation of 0.92. Nonfungal sequences comprised less than 0.5% of the soil data set, with most attributable to vascular plants. Our results suggest that high-throughput methods can produce fairly accurate estimates of fungal abundances in complex communities. Further improvements might be achieved through corrections for rRNA copy number and utilization of standardized mock communities.

**IMPORTANCE** Fungi play numerous important roles in the environment. Improvements in sequencing methods are providing revolutionary insights into fungal biodiversity, yet accurate estimates of the number of fungal species (i.e., richness) and their relative abundances in an environmental sample (e.g., soil, roots, water, etc.) remain difficult to obtain. We present improved methods for high-throughput Illumina sequencing of the species-diagnostic fungal ribosomal marker gene that improve the accuracy of richness and abundance estimates. The improvements include new PCR primers and library preparation, validation using a known mock community, and bioinformatic parameter tuning.

## INTRODUCTION

Fungi play key roles in the environment, with particular importance in nutrient cycling and modulation of plant growth (1). Estimates of global fungal species richness are in the millions (2–4), while <2% have been formally described. Fungal taxa that have been subject to detailed study display complex biogeographic patterns and histories; most species do not have global distributions (5). Fungi are also highly responsive to environmental influences, including global change factors, such as N deposition (6) and temperature (7). Thus, increased knowledge of the scope, structure, and dynamics of fungal biodiversity on Earth is urgently needed. High-throughput sequencing methods are offering deeper insight into fungal biodiversity (4, 8–10), yet current methods provide relatively poor estimates of total species richness and abundances of constituent taxa due to biases and error introduced during DNA extraction, PCR, sequencing, and bioinformatic analyses (11–18).

Pyrosequencing on the 454 platform has been the predominant approach for fungi (19), but many studies are now utilizing the shorter reads but greater sequencing depth available with the Illumina platform (20). While these approaches offer unprecedented access to biodiversity (10, 21), there is also voluminous literature concerning the many artifacts and biases that attend these approaches (e.g., 18, 22–25). Analyses of known microbial templates, i.e., “mock communities,” have proven particularly informative with respect to diagnosing and improving problems arising at both the bench and the bioinformatic stages of analysis (18, 26). So far, only a few studies have utilized mock communities for fungal research (12, 27–30). A particularly problematic issue in fungal ecology has been the accurate estimation of fungal abundances. For example, Amend et al. obtained over an order of magnitude in difference between input numbers of fungal spores and resulting read abundance in mixed communities using a 454 approach (31). Bokulich and Mills analyzed the relationship between starting genomic DNA abundance, corrected for ribosomal copy number, and resulting read number on the Illumina platform and reported relatively poor correspondence that was primer specific (28). So far, only one study of which we are aware recovered relative taxon read abundances that were strongly related to the relative input template abundances in a fungal mock community study; in that case, the templates were PCR amplicons rather than genomic DNAs of the target organisms, thus representing a simplification of real-world samples (27). Although there are many potential causes for poor correlations between observed and expected abundances, there are three leading explanations. First, copy numbers for the tandemly repeated nuclear ribosomal operon vary widely among fungi, from tens (32) to hundreds (33, 34) of copies. Second, PCR biases that may be due to features of the intervening amplicon, such as length and secondary structure, and/or imperfect matches between primers and template, are known to distort read abundances (27, 35). Third, the efficiency of DNA extraction can vary among fungal taxa and cell types (31).

Most of the fungal studies described above have utilized portions of the nuclear ribosomal internal transcribed spacer (ITS) region (ITS1-5.8S-ITS2) because it is the approved fungal barcode (36) and has been used very effectively in fungal ecology for 25 years (37). A number of primer options exist for accessing various parts of the ITS and surrounding ribosomal coding regions (27, 28, 37–40). In the context of ITS amplicon sequencing on the Illumina platform, we view several primer attributes as important: PCR efficiency, coverage, selectivity, and variation in amplicon size. With respect to coverage, the ideal is to amplify all species and lineages of Fungi. With respect to selectivity, there are two options. One option is to attempt to design primers with mismatches to other major lineages of eukaryotes to reduce their amplification. The second option is to design broad-spectrum primers and accept amplification of nonfungal lineages, with removal or segregation of these reads at the bioinformatic stage. Depending on the type of substrate and study goals, either approach may be preferable. However, in our experience, it is often very difficult and time-consuming to distinguish sequences belonging to certain microbial eukaryote lineages, such as the Cercozoa, from fungal sequences. Furthermore, certain substrates, such as leaves or roots, have very high concentrations of nonfungal eukaryotic DNA, meaning that a very large fraction of the reads would be discarded when using nonselective primers. Hence, for working with soils, plant tissues, and other substrates where fungi may be a minority, selective primers may prove advantageous. We found that no previously published fungal ITS primers appropriate for the Illumina platform satisfied all of the above-mentioned criteria. We designed two new primers targeting the ITS2 region and tested them using mock communities and previously sequenced soils on the Illumina MiSeq platform.

## MATERIALS AND METHODS

### Primer design.

We focused primer design efforts on the 5.8S and the 5′ region of the nuclear ribosomal large subunit (LSU) because we wished to target ITS2 and thereby avoid the intron insertion site that occurs at the 3′ end of the nuclear ribosomal small subunit (SSU). This intron appears to be easily gained and lost and mutates rapidly. The intron has been reported in diverse members of the Ascomycota (41–47) and can be up to 400 bp in length, thus pushing ITS1 amplicons to over 600 bp, which would likely bias against the detection of these taxa on the Illumina platform. Inspection of the 3′ end of the SSU suggested that it would be impossible to design a fungus-selective primer downstream of the intron insertion site, making ITS1 a less-preferred target. To design primers targeting ITS2, we assembled a broad set of fungal and nonfungal 5.8S and LSU sequences, starting with alignments made available by the All Fungal Tree of Life (AFTOL) Consortium (48). We augmented the 5.8S and LSU alignments with additional sequences to represent other major lineages of eukaryotes: Alveolata, Amoebozoa, Apusozoa, Cryptophyta, Ichthyosporea, Haptophyceae, Annelida (Metazoa), Anthozoa (Metazoa), Arthropoda (Metazoa), Bilateria (Metazoa), Chordata (Metazoa), Cnidaria (Metazoa), Mollusca (Metazoa), Nematoda (Metazoa), Platyhelminthes (Metazoa), Rotifera (Metazoa), Tardigrada (Metazoa), Rhizaria, Chlorophyta (Viridiplantae), and Streptophyta (Viridiplantae). The alignments were inspected visually for regions that appeared to be conserved across fungi yet had mismatches with other eukaryotes. Prospective sites were then evaluated for melting temperature, hairpins, self, and cross-dimers using OligoAnalyzer (https://www.idtdna.com/calc/analyzer); prospective primers with problematic attributes were discarded. Because the 5.8S region is only ∼165 bp, options for primer locations are limited. However, we were able to identify one promising primer in the 5.8S, named 5.8S-Fun, and one in the LSU, named ITS4-Fun. The ITS4-Fun primer location partially overlaps the previously published universal primer ITS4 (49).

To further evaluate previously published and new primers, we estimated specificity, coverage, and other features analytically using PrimerProspector version 1.01 (50). We evaluated deep phylogenetic coverage and selectivity of 5.8S-Fun and fITS9 (27) using the same AFTOL 5.8S alignment described above. We then evaluated coverage across Fungi in more detail using the UNITE 97% identity ITS species hypothesis data set derived from all fungal ITS sequences in GenBank (51) and compared ITS4-Fun with the universal primer ITS4 using the SILVA LSU database (52). We used the taxa_coverage.py script in PrimerProspector to calculate the percent coverage by taxon for each primer at a range of taxonomic levels (domain to family). Potential amplification was evaluated using the default weighted scoring scheme, which applies a stronger penalty to 3′ mismatches. Barcoded PCR primer constructs were tested with PrimerProspector's check_primer_barcode_dimers.py script, using −20 kcal/mol as a threshold for discarding potential constructs with significant secondary structures or dimers.

While type I self-splicing introns downstream of popular ITS primers in the SSU rRNA gene have been reported from several Ascomycota (41–44, 46, 47), their incidence in natural communities has not been analyzed, to our knowledge. Thus, in order to evaluate the frequency and length characteristics of these introns in a natural sample of soil fungi, we obtained OTU representative sequences published by Taylor et al. (4). Because this large data set was obtained by Sanger sequencing of clone libraries, the data should be less subject to size biases than with most next-generation sequencing methods, such as 454 and Illumina. OTU sequences were aligned using MAFFT in a multistep approach using a series of blocks in order to obtain high-quality alignment of the conserved small subunit region at the beginning of the fragment and 5.8S regions in the middle of most fragments. We filled in missing bases in cases where only a few conserved bases upstream of the intron insertion site were missing in order to obtain exact fragment lengths. OTUs for which the entire SSU or 3′ end of the 5.8S regions were missing were excluded from further comparisons (we refer to these as ambiguous OTUs). OTUs containing introns were easily identified by eye by virtue of the interruption of the conserved bases at the 3′ end of the SSU. OTUs containing definite introns were then compared to OTUs lacking obvious introns in the SSU insertion site. We compared the richness of OTUs in both groups, the taxonomic distribution of intron-containing taxa, and the relative abundances of sequences belonging to these OTUs utilizing the species × site matrix from the study by Taylor et al. (4). The means and ranges of ITS1 amplicon lengths were estimated, after excluding OTUs that were not complete on the SSU or 5.8S end of the fragment.

We also used this data set to calculate the range, mean, and standard deviations of amplicon sizes when using primers ITS1-F with ITS2 to amplify the ITS1 region versus our new primers to amplify the ITS2 region.

### Mock community and soil template DNAs.

Low-diversity but phylogenetically broad mock communities were created as follows. Amanita muscaria 3-1-B2-1-2s (Basidiomycota, isolated from Alaskan fruitbody), Amphinema byssoides R-NC03 (Basidiomycota, isolated from Picea abies ectomycorrhiza in Finland), Coprinopsis cinereus (Basidiomycota, obtained from the Fungal Genetics Stock Center, strain FGSC 9003), Mortierella alpina (Mucoromycotina, obtained from the American Type Culture Collection, strain 42430), Spizellomyces punctatus (Chytridiomycota, obtained from the American Type Culture Collection, strain 48900), Tricholoma vaccinum 18-1-B1-A1-2s (Basidiomycota, isolated from Alaskan fruitbody), and Tylospora asterophora R-MF02 (Basidiomycota, isolated from Picea abies ectomycorrhiza in Finland) were grown in modified Melin-Norkrans (MMN) broth for up to 6 months on a rotary shaker at room temperature. Mycelium was harvested by filtration through cheesecloth, freeze-dried, and then ground in liquid nitrogen with a mortar and pestle. Genomic DNAs were isolated using the Qiagen Genomic-tip kit. Genomic DNA from Schizosaccharomyces pombe strain 972 h- was provided by the Broad Institute. Genomic DNA concentrations were estimated by fluorescence on a NanoDrop 3300 using PicoGreen (Quant-iT kit; Invitrogen) with lambda DNA standards. The averages of three NanoDrop 3300 readings were used for calculations. The 5.8S-Fun/ITS4-Fun amplicons (including core primers but not adaptors) for these eight taxa range from 396 to 514 bp (mean, 440 bp). Two “tiered” mock communities were created wherein taxa were randomly assigned to high, medium-high, medium-low, and low relative abundances (see Table S1 in the supplemental material). These abundances spanned three orders of magnitude (0.043% to 43%). Different taxa were assigned to the 4 abundance levels in the two communities, designated mock A and mock B. Both mock communities had final concentrations of 11.2 ng/μl.

In order to evaluate the performance of our primers on a more complex real-world fungal community, we also analyzed five soil DNA extracts that have been extensively analyzed using large-scale Sanger sequencing of ITS-LSU clone libraries (4, 53). These boreal forest soil DNAs were extracted using the Mo Bio PowerMax kit (Mo Bio Laboratories, Carlsbad, CA, USA) and normalized to 2.5 ng/μl. The TKN sample is from a lowland black spruce (Picea mariana) forest, UP1 samples are from early stage upland mixed forest, and UP3 samples are from a late-stage upland white spruce (Picea glauca) forest. Samples with an “O” are from the organic horizon, while samples labeled “M” are from the mineral horizon. The final number indicates the collection year, e.g., 2004. Detailed descriptions of these sites and samples are in references 4, 53, and 54.

### PCR and sequencing.

We utilized a one-step amplification protocol in which the core PCR primer, indexes, linkers, and Illumina sequencing adaptors were included in a single oligonucleotide. The indexes used were 12-bp Golay barcodes (55). The Illumina forward adaptor and barcodes were added to the ITS4-Fun primer rather than the 5.8S-Fun primer to avoid excessive hairpin formation. Thus, the forward reads obtained from the Illumina sequencing are in reverse orientation with respect to the ribosomal operon. The oligonucleotide sequences were (core PCR primer in bold) 5.8S-Fun (5′-CAAGCAGAAGACGGCATACGAGAT-NNNNNNNNNNNN-AGTCAGTCAG-GG-**AACTTTYRRCAAYGGATCWCT**-3′) and ITS4-Fun (5′-AATGATACGGCGACCACCGAGATCTACAC-TATGGTAATT-AA-**AGCCTCCGCTTATTGATATGCTTAART**-3′). From 5′ to 3′, each oligonucleotide includes (i) the 24- to 29-bp Illumina sequencing adaptor, (ii) the 12-bp Golay barcode (5.8S-Fun only), (iii) a 10-bp primer pad, (iv) a 2-bp linker, and (v) the 21- to 27-bp core primer. Components i to v are separated by dashes in the sequences above, and Golay barcode bases are shown as Ns. PCRs were carried out in 25-μl reaction mixtures with 5 μl of template DNA (mock community or soil) using illustra PuReTaq ready-to-go PCR beads (GE Healthcare Life Sciences, Pittsburgh, PA, USA), with 15 replicates per sample, and using a different Golay index for each sample. The thermocycling conditions were initial denaturing at 96°C for 2 min, followed by 27 cycles of denaturation at 94°C for 30 s, 58°C for 40 s, 72°C for 2 min, and a final extension at 72°C for 10 min. Thermocycling was carried out in MJ PTC-200 instruments.

The replicate PCRs were pooled, cleaned with Zymo-5 columns (Zymo Research, Irving, CA, USA), and then quantified on a NanoDrop 3300 with PicoGreen. The two mock community samples and five soil samples were then combined, and any remaining short fragments were removed by size fractionation over a ChromaSpin 200 column (BD Biosciences, San Jose, CA, USA).

Libraries were quantified using adaptor-specific probes on an Illumina Eco quantitative PCR (qPCR) instrument.

Sequencing of these libraries was carried out in May 2013 using Illumina 2 × 250-bp extra-long read kit on a full run of the MiSeq instrument. PhiX control oligonucleotides were spiked in to the run to add base diversity.

### Sequence processing.

Reads were assigned to samples, and adaptors, indexes, and primers were marked using Picard (https://broadinstitute.github.io/picard/index.html), resulting in bam files that were converted to fastq using bam2fastq (https://gsl.hudsonalpha.org/information/software/bam2fastq). Overall sequencing quality was evaluated visually using FastQC (http://www.bioinformatics.babraham.ac.uk/projects/fastqc/). The majority of subsequent analyses were conducted in QIIME 1.9.1 (56). Initial quality filtering was carried out using the split_libraries_fastq.py script with strict settings (all scripts and settings are listed in Table S2 in the supplemental material). We found that there was insufficient high-quality sequence for all but a small fraction of forward and reverse reads to be joined. Forward read data (originating from the ITS4 end of the amplicon) were slightly higher in average quality and, hence, all analyses were carried out with forward read data only. We also discovered that some phiX174 genomic reads were not removed by the Illumina software. Therefore, all data were run through ITSx to identify and retain only reads with features signifying likely origins as eukaryotic ITS sequences (57). Putative chimeric sequences were identified using the *de novo* method in USEARCH6.1 (58, 59).

Sequences were clustered into OTUs using pick_open_reference_otus.py in QIIME. Clustering was carried out independently for mock A, mock B, and the five combined soil samples. For mock communities, full-length Sanger sequences for the mock community members were provided as the reference database (i.e., as seeds for subsequent clustering). For the soil samples, the complete UNITE 97% species hypothesis database formatted for QIIME (ver7_97_01.08.2015) was provided as the reference database. For the mock community data sets, we evaluated the influence of the clustering algorithm, namely, UCLUST (58), USEARCH6.1 (58), and Swarm v1 (60), and different identity/distance parameter values (d = 1 to d = 5 for Swarm; similarity, s = 93, 95, and 97 for UCLUST and USEARCH6.1). USEARCH6.1 with s of 93 was chosen and used for analyses of the soil data. We also compared *de novo* to open reference clustering for the mock communities due to concerns about the open reference method raised by Wescott and Schloss (61).

We used the assign_taxonomy.py script with the BLAST method to match the representative sequences for each OTU (maximum E value, 0.001) to the UNITE database. Because a number of OTUs returned no matches compared with the UNITE database, we also conducted separate blastall (62) searches against the entire NCBI nucleotide database. We list UNITE matches where available and NCBI top hits otherwise in Data Set S1 in the supplemental material. We used biom_convert (63) to convert the BIOM-formatted OTU tables from QIIME to tab-separated files of OTU abundances by sample to facilitate analysis in other programs.

### Statistical analyses.

The expected numbers of reads for each taxon in a mock community were calculated by multiplying their percent genomic DNA contribution to the pooled sample by the total number of passing fungal reads obtained for that community. We calculated the Pearson correlation between the observed and expected numbers of reads and tested the significance of the relationship by simple linear regression. We tested for deviation from expected counts using a contingency table chi-square analysis.

The correspondence between fungal community composition derived from Sanger sequencing of clones of the 1,200- to 1,500-bp ITS1-FL/TW13 amplicon (53) versus the 100- to 200-bp ITS2 reads derived using the new primers on the Illumina MiSeq was analyzed in three ways. First, we carried out cluster analyses using average linkage applied to Bray-Curtis abundance-based distance matrices for each data set independently. The clustering of sites was then compared visually. Second, we carried out Mantel tests for correlation between the two distance matrices, with significance determined by permutation (*n* = 10,000). For all analyses, the “general relativization” option was used to equalize sequencing effort across samples, although results were nearly identical without this transformation (data not shown). All multivariate analyses were conducted in PC-ORD version 5 (64). Third, we compared the identities of the dominant OTUs from the two methods as follows. Because it utilizes a rigorous full-alignment clustering approach, we clustered the 20 most abundant Sanger OTUs with all MiSeq OTUs at 97% identity using CAP3 (65) and then compared the abundance ranks of these matched OTUs. Due to different sequence lengths, it would have been inappropriate to include both Sanger and Illumina sequences in one USEARCH clustering step.

### Accession number(s).

The new Illumina sequences have been submitted to the NCBI Sequence Read Archive; the accession numbers are provided in Table 1.

## RESULTS

### *In silico* analyses.

**FIG 1:**
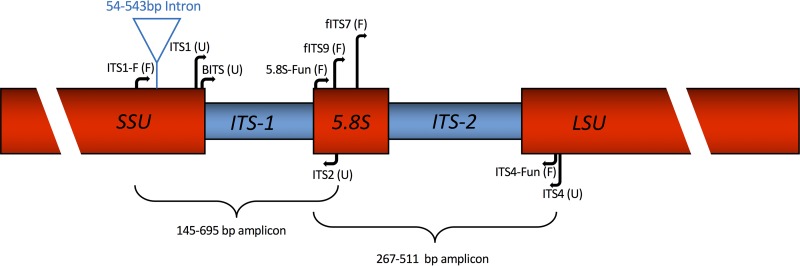
Fungal nuclear ribosomal ITS primer map. Blocks in red are the SSU (18S or small subunit nuclear rRNA gene), 5.8S (also a structural RNA gene), and LSU (28S or large subunit nuclear rRNA gene). The blue triangle above the SSU gene indicates the type I self-splicing intron insertion site. The two transcribed but nonstructural spacer regions, ITS-1 and ITS-2, are shown in blue blocks. Primer names and relative positions are given. In parentheses, F indicates primers that were designed to select against nonfungal taxa; U indicates primers that were designed as universal eukaryote primers. While designed to be selective for Fungi, ITS1-F has the potential to amplify a range of protist lineages (see Fig. S2 in the supplemental material). Primers fITS7 and fITS9 were designed to be fungal selective but have the potential to amplify diverse plants, according to our *in silico* analyses and empirical reports of the authors (27). Primers fITS9 and ITS2 are unlikely to amplify some important fungal lineages, according to our *in silico* analyses (see Fig. S3 in the supplemental material). At the bottom are estimates of the range of amplicon lengths, including taxa with and without the 3′ SSU intron, based on the ITS-LSU OTU representative sequences from reference 4. Gene sizes and primer positions are not to scale.

**FIG 2:**
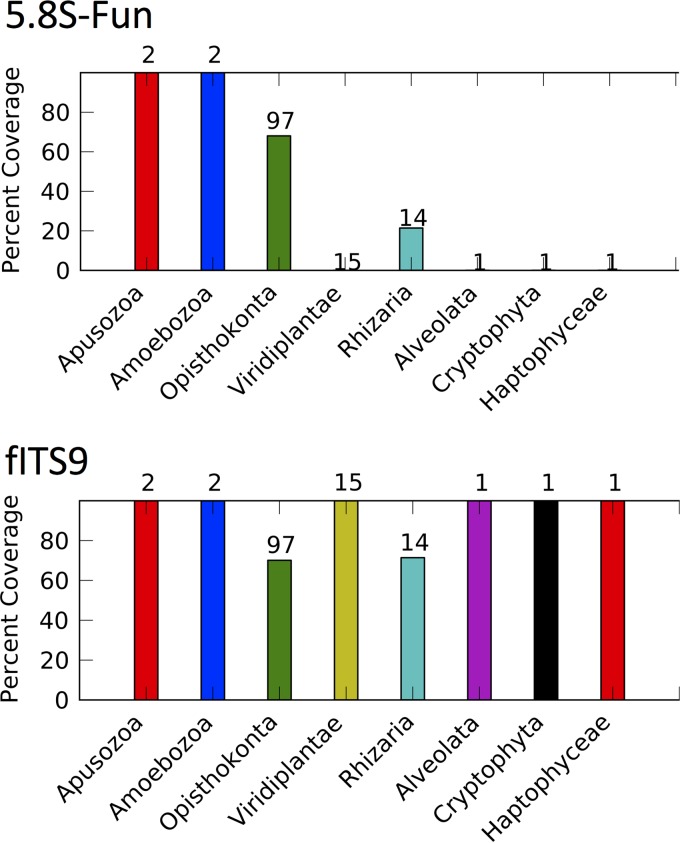
Each column shows the proportion of taxa (*y* axis, percent coverage) within a given deep-level lineage that are predicted by PrimerProspector to be amplified using the specified PCR primer. The kingdom Fungi falls within the Opisthokonta; both primers display similar coverage of Opisthokonta at this coarse phylogenetic level. However, fITS9 is also predicted to amplify members of Viridiplantae (land plants), Rhizaria, Alveolata, Cryptophyta, and Haptophyta, while 5.8S-Fun is not predicted to amplify the representatives of these lineages. 5.8S sequences from AFTOL were provided as input.

Despite the numerous criteria we wished to meet and the short regions available for primer location, we were able to design improved primers targeting the fungal 5.8S and 5′ LSU for amplicon-based Illumina community profiling, as depicted in Fig. 1. Visual inspection of the 5.8S and LSU alignments suggested that the new primer 5.8S-Fun would have wider coverage across Fungi yet stronger selectivity, particularly against plants, than existing primers, including ITS3 (49, 66) and fITS9 (27). Detailed taxonomic analyses with PrimerProspector support these expectations (Fig. 2; see also Fig. S3 in the supplemental material). For example, fITS9 will theoretically amplify all Viridiplantae, as well as various algal and protist lineages (Fig. 2). In contrast, 5.8S-Fun is a poor match to the analyzed Viridiplantae and most eukaryote sequences. We did not specifically evaluate fITS7, because this primer has been noted to exclude certain Ascomycota (Penicillium, Orbiliales), most Mucorales (27), and is a poor match to many Glomeromycota (E. A. Lilleskov, unpublished data). PrimerProspector analyses reveal high coverage of 5.8S-Fun across nearly all orders of Fungi available in the large UNITE database (see Fig. S3 in the supplemental material). fITS9 also has wide coverage at the level of fungal order, although a few groups exhibit lower coverage than 5.8S-Fun (see Fig. S3). We note that fITS9 also has strong secondary structure that may decrease amplification efficiency. ITS4 is commonly used in fungal studies (27, 38) but was not designed to be fungus selective (49). By moving the primer upstream 6 bases, we were able to access a site that remains highly conserved across Fungi but differs from plants. These observations are supported by PrimerProspector analyses using the Silva large subunit database: ITS4-Fun had nearly 100% coverage for Fungi but lower coverage for some other lineages, particularly Viridiplantae.

In our analyses of soil amplicon Sanger sequences from Taylor et al. (4), we found 148 OTUs out of a total of 990 unambiguous OTUs that had an SSU intron. Thus, intron-containing OTUs comprised 15% of the species richness in this particular soil data set. These intron-containing OTUs comprised 6.7% of the 28,775 clone sequences. Introns were particularly abundant in OTUs assigned to the Helotiales (63 OTUs) and Chaetothyriales (20 OTUs) of the Ascomycota. However, they were widely distributed across the Pezizomycotina, with evidence for SSU introns in 19 orders (see Table S3 in the supplemental material). We found evidence for an intron in one OTU assigned to the Basidiomycota (Sebacina sp.). The length from ITS1-F to the start of the 5.8S ranged from 95 to 514 bp (mean ± standard deviation [SD], 229.2 ± 39.9 bp) in OTUs lacking the SSU intron [although the 514-bp OTU may have intron(s) in other locations]. In contrast, the equivalent lengths for intron-containing OTUs ranged from 250 to 645 bp (mean ± SD, 478.8 ± 89.9 bp). Thus, intron-containing taxa have a 250-bp-longer ITS1 region than non-intron-containing taxa, on average. A truncated alignment of the 3′ end of the SSU in exemplary intron-lacking and intron-containing OTUs is shown in Fig. S1 in the supplemental material.

The same clone library data set was used to evaluate overall ITS1 versus ITS2 amplicon lengths, regardless of introns. Amplicons spanning the region targeted by the primers ITS1-F and ITS2 were shorter, with a mean length of 309.4 bp, but had higher length variation, with a range from 145 to 695 bp and standard deviation of ±94.8 bp. In contrast, the ITS2 amplicon predicted when using 5.8S-Fun with ITS4-Fun ranged from 267 to 511 bp, with a mean of 394.2 bp and a standard deviation of ±36.6 bp.

### Sequence quality and processing.

On average, read quality decreased rapidly after about 120 bases, as was typical when the MiSeq 500-cycle paired-end chemistry was first released. On average, only 15% of the reads passed the initial quality filtering in split_libraries_fastq.py, with the majority of reads discarded as too short after truncation (Table 1). The average read length after filtering ranged from 201 to 211 bases (Table 1). A very small fraction of reads (0.021%) were discarded as putative chimeras (Table 1). ITSx identified putative ITS2 sequences in the vast majority of reads that were retained after split_libraries_fastq.py (Table 1); however, this step did effectively remove all phiX174-related OTUs.

**TABLE 1:** Sequence processing metrics

Sample	SRA sample	SRA experiment	SRA run	Total no. of reads	No. of reads retained after QC^*a*^	Median read length (bases)	No. of reads retained after ITSx	No. of chimeric reads identified	No. of OTUs^*b*^
Mock.A	SRS1648698	SRX2056713	SRR4070107	972,843	76,842	201	76,244	8	21
Mock.B	SRS1648691	SRX2056697	SRR4070093	1,025,155	200,436	208	199,600	35	21
TKN0051.O.4	SRS1648565	SRX2056570	SRR4069943	2,125,478	304,383	204	297,784	71	1,349
UP1B.M.5	SRS1648566	SRX2056569	SRR4069942	1,758,453	261,735	211	252,437	49	1,339
UP1B.O.5	SRS1648564	SRX2056568	SRR4069941	1,060,118	190,173	207	184,451	55	795
UP3A.M.5	SRS1648527	SRX2056531	SRR4069914	1,526,506	222,749	207	218,855	59	764
UP3A.O.5	SRS1648182	SRX2056187	SRR4069933	1,401,263	224,421	206	219,897	29	1,187
Total				9,869,816	1,480,739		1,449,268	306	2,632

a QC, quality control.

b With abundances of ≥2, i.e., global singletons were removed. Some of the same OTUs occur in multiple samples, hence, the total value in the last row is less than the cumulative total of the other rows.

### Mock community analyses.

Depending on the stringency of read quality control in split_libraries_fastq.py and the clustering method, we recovered from 21 to >13,000 OTU per mock community. In particular, we found the retention of shorter reads and reads with Ns or strings of lower-quality bases led to OTU inflation. BLAST searches revealed that numerous OTUs were clustered around the expected mock community member Sanger sequence. Thus, it appears that sequencing error contributed to OTU inflation under relaxed settings. We also found that UCLUST and Swarm resulted in many more spurious OTUs than USEARCH6.1 under a range of parameter settings. Again, the diagnosis of spurious OTUs was based on the recovery of large numbers of distinct OTUs most closely related to a single mock community member sequence. With strict quality filtering and a similarity threshold of 93%, we recovered close to the expected numbers of OTUs.

In both mock communities, all 8 expected community members were recovered, despite starting genomic concentrations varying over 3 orders of magnitude (see Table S1 in the supplemental material). For mock A, a total of 76,199 reads passed the quality control steps and were clustered into the 21 OTUs with abundances of 2 or greater (Table 1; see also Table S4 in the supplemental material). The 8 mock community members were split into 12 OTUs; taxa with multiple OTUs were A. byssoides (3 OTUs) and C. cinerea (2 OTUs). Fungi that were not intentionally included as mock community members, i.e., contaminants, contributed 9 additional OTUs. However, only 0.03% of the fungal reads originated from contaminant fungi. For mock B, a total of 199,559 reads passed the quality control steps and were clustered into 21 OTUs with abundances of 2 or greater (Table 1; see also Table S4). The 8 mock community members were again split into 12 OTUs; taxa with multiple OTUs were A. byssoides (3 OTUs) and T. asterophora (2 OTUs). Again, 9 OTUs were attributed to contaminant fungi; 0.05% of the reads originated from contaminant fungi. The contaminants recovered from mock A and mock B had only one OTU in common. The results using *de novo* rather than open reference clustering were nearly identical: the same mock OTUs were recovered, with a maximum of 9 greater or fewer reads for a mock community member in comparing the two methods (a difference of <0.2% in read abundances). Interestingly, two additional mock communities were oversplit (multiple OTUs rather than one) for mock A using *de novo* clustering; the same two community members were oversplit with both methods for mock B.

**FIG 3:**
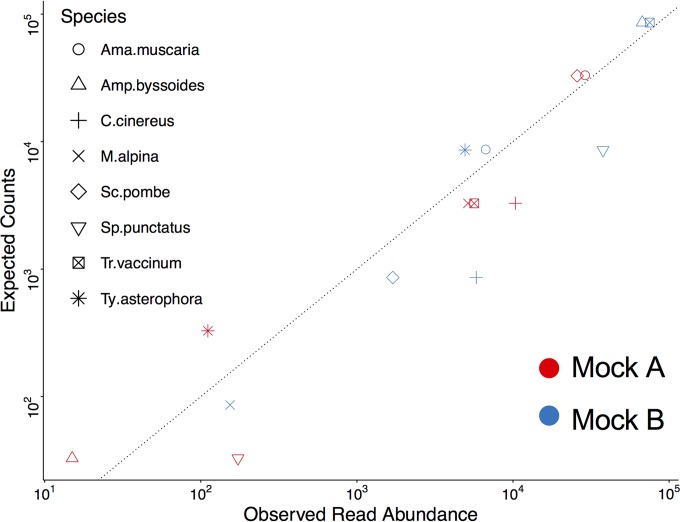
Relationship between expected and observed mock community member abundances. The expected numbers of reads (counts) for a given mock community taxon are given on the *y* axis, based on the proportion of the total mock community genomic DNA contributed by a particular taxon multiplied by the total number of reads obtained. The *x* axis represents the actual observed read abundance for each mock community taxon. Mock community A data points are illustrated with red symbols, while mock community B data points are shown in blue symbols. Each mock community taxon appears twice on the graph (the same symbol in red and blue), because they were added at different relative abundances in the two mock communities. The dashed line equals the observed trend line. Ama.muscaria, *Amanita muscaria*; Amp.byssoides, Amphinema byssoides; Sc.pombe, Schizosaccharomyces pombe; Sp.punctatus, Spizellomyces punctatus; Tr.vaccinum, Tricholoma vaccinium; Ty.asterophora, Tylospora asterophora.

There was a strong correlation between observed and expected numbers of reads for mock community members (mock A, *r* = 0.97, *P* = 0.0001; mock B, *r* = 0.94, *P* = 0.0006; Fig. 3). However, there were also deviations between the observed and expected values, resulting in a significant chi-square (*P* = 0.0003). Interestingly, some of these patterns were consistent across the two libraries with taxa added at different abundances. For example, S. punctatus yielded 4.4 and 5.3 times greater numbers of reads than expected, C. cinereus produced 3.1 to 6.8 times more reads than expected, and T. vaccinum produced 1.7 to 3 times fewer reads than expected. A. muscaria also consistently gave fewer reads than expected. This result is unlikely to be explained by length bias, as amplicon length was intermediate in C. cinereus (413 bp) and long in S. punctatus (451 bp).

### Soil analyses.

We obtained from 184,451 to 297,784 reads per soil sample that passed the ITSx step (Table 1). Chimera filtering and clustering resulted in 764 to 1,349 OTUs per sample. Phylogenetic representation of the OTUs was broad: we obtained OTUs attributed to Dikarya, Glomeromycota, and several basal fungal lineages, including the Mucoromycotina, Rozellomycota, and Chytridiomycota (Table 2).

**TABLE 2:** Read abundances by phylum for the five soil samples combined

Sequences	No. of OTUs	Percent OTUs^*a*^	No. of reads	Percent reads^*a*^
Ribosomal sequences				
Fungi				
Ascomycota	1,152	43.79	282,048	24.195
Basidiomycota	1,272	48.35	867,450	74.414
Chytridiomycota	26	0.99	141	0.012
Entorrhizomycota	1	0.04	2	0.000
Glomeromycota	32	1.22	1,089	0.093
Mortierellomycotina	50	1.90	7,915	0.679
Mucoromycotina	30	1.14	6,389	0.548
Rozellomycota	5	0.19	70	0.006
Unassigned fungi	63	2.39	602	0.052
Subtotal	2,631		1,165,706	
Nonfungi				
Rhodophyta	2		5	0.000
Stramenopiles	1		5	0.000
Viridiplantae	25		3,713	0.317
Nonribosomal sequences	225		2,707	0.231
Total			1,172,136	

a Percentages for fungal phyla were calculated without nonfungal sequences.

The vast majority of reads from soil samples were identified by ITSx as containing a eukaryotic ITS2 full or partial sequence; of these, 96.6% were attributed to the kingdom Fungi (Table S5 in the supplemental material). However, when we attempted to identify all OTUs by BLAST searches, the only nonfungal lineages recovered were Rhodophyta, Stramenopiles, and Viridiplantae, which comprised only 0.32% of the reads (Table 2). Hence, either ITSx misattributed fungal sequences to other lineages, such as the Metazoa, Amoebozoa, and Rhizaria, or these nonfungal reads were singletons and were eliminated at the clustering step. Viridiplantae dominated the nonfungal ribosomal OTUs, with many perfect matches to taxa found at these sites, including Chamerion angustifolium, Mertensia sibirica, Vaccinium vitis-idaea, Betula pendula, Rhododendron tomentosum, Alnus viridis, and Calamagrostis canadensis (see Data Set S1 in the supplemental material).

Because we compared only the results from clone library Sanger sequencing of the ITS1-F/TW13 ITS-LSU amplicon with Illumina sequencing of the 5.8S-Fun/ITS4-Fun amplicon for five soil samples, ordination was not informative for a comparison of patterns of community composition. Cluster analysis was deemed more useful and resulted in identical clustering of samples (see Fig. S4 in the supplemental material). In both cases, the organic and mineral horizon samples from the same site clustered together, and the upland sites formed a group distinct from the lowland black spruce sample. These results mirror those seen in a larger study that included the same samples (53). In a more rigorous comparison, a Mantel test yielded a strong statistically significant correlation between the Illumina and Sanger data sets (standardized Mantel statistic, *r* = 0.92, *P* = 0.008). The Mantel statistic ranges from −1 for completely opposed patterns of intersample distances to +1 for identical patterns of intersample distances.

Searches for representative sequences from the two data sets that clustered together at ≥97% revealed considerable overlap in the dominant taxa recovered. Among the 20 most abundant taxa recovered from clone library Sanger sequencing, all were identified within the top 51 OTUs in the much larger MiSeq data set, with one exception: an Archaeorhizomyces OTU with rank 11 in the Sanger data set was also present in the MiSeq data set but with rank 221. This difference may be due to primer bias. Four bases at the 5′ end of ITS4-Fun do not match available Archaeorhizomyces sequences. However, the 19 consecutive 3′ bases of ITS4-Fun are a perfect match.

## DISCUSSION

We view the frequency of SSU introns in our Sanger-sequenced soil clone libraries as an underestimate. While cloning and Sanger sequencing are less subject to the extreme biases against longer amplicons seen in 454 and Illumina sequencing technologies, there is likely to be some bias toward shorter fragments at both the PCR and cloning steps. As such, our finding that 15% of the recovered fungal species in these soils were intron containing implies that the fungi comprise a significant component of these communities. The mean ITS1-F to ITS2 amplicon length of ∼530 bp predicted for these fungi suggests that they will be strongly underrepresented when using current Illumina technology. The intron first described in the Pneumocystis jirovecii nuclear small subunit RNA gene was 390 bp and displayed a splice site, conserved domains, and secondary structure typical of well-characterized group I self-splicing introns (41). The putative introns detected in our data set averaged 291 bp in length and were usually positioned between conserved U and G residues, the expected slice junctions. We did not further evaluate these sequences, as it is beyond the scope of the current study. It is suspected that self-splicing group I introns may propagate by horizontal gene transfer (67), which would obscure their phylogenetic signal and utility in taxonomic placement. The finding of a putative intron in a distantly related basidiomycete supports this contention. This is another reason why sequencing an ITS1 fragment spanning this intron site may undermine studies of fungal communities.

For applications in which it may be desirable to cast the widest possible taxonomic net for fungi and yet reduce sequencing of nonfungal eukaryotes, such as studies of mycorrhizae, plant endophytes, or soil, the primers presented here offer desirable features with respect to coverage and selectivity relative to other ITS primers available. 5.8S-Fun displayed wide coverage and strong selectivity *in silico*, and we detected no bias against basal fungal lineages in our mock community analyses. When 5.8S-Fun was paired with the fungus-selective primer ITS4-Fun, we obtained relatively few nonfungal sequences from soil samples (0.3%). By way of comparison, the venerable fungus-selective primer ITS1-F performs very well with respect to minimizing the amplification of plant genes and has been the workhorse in mycorrhizal ecology for over 20 years (37). However, ITS1-F is upstream of the SSU intron insertion site. Taxa containing this intron will likely fail to be detected in high-throughput sequencing studies using ITS1-F due to the much longer amplicon produced. Furthermore, ITS1-F is a perfect match to a number of protist lineages commonly encountered in soil (see Fig. S2 in the supplemental material) and so can retrieve many difficult-to-place nonfungal OTUs. ITS1-F also has mismatches with various fungi (30). The newer primer fITS9 (27) is a strong match to plants, while the primer BITS introduced by Bokulich and Mills was not designed to exclude nonfungal lineages (28). We were able to design over 192 adaptor-barcode-linker-primer oligonucleotide combinations in PrimerProspector by using ITS4-Fun as the forward primer and 5.8S-Fun as the reverse primer (see Data Set S2 in the supplemental material). These oligonucleotides were effective in a single-index one-step protocol, as utilized in this study. We have also successfully used these core primers in a dual-index two-step protocol (68).

Our study underlines the fact that the same raw sequence data can yield extremely different perspectives, depending on the exact bioinformatic steps (12, 69). For our mock community data, we obtained from 21 to >13,000 OTU, depending on the quality filtering and clustering settings. Many of these OTUs were due to phiX174 sequences prior to screening with ITSx. However, we still obtained thousands of mock community OTUs after ITSx filtering when the base call quality filtering was not stringent or a narrow clustering method and threshold were used (e.g., UCLUST at 97%). Even with a relatively low percent identity clustering threshold (93%) with USEARCH, we still obtained up to 3 OTUs stemming from the same input mock community taxon. With our current data, it is impossible to say whether this OTU inflation was due to PCR and sequencing error or to real intraindividual variation in ITS sequences across the rRNA repeat. The levels of rRNA gene polymorphism within individuals and species remain somewhat controversial in fungi (70), although at least one high-throughput sequencing study suggests that this should not be a major problem for many fungal taxa (71). While 93% identity is far below the widely used species-level threshold of 97%, it is important to note that USEARCH uses a complete-linkage clustering approach (58), which produces much smaller (i.e., more numerous) clusters than the more widely used single-linkage approach (69). Thus, clustering algorithm and software can have just as much impact on estimated richness as the percent identity threshold. Therefore, percent identity thresholds should not be viewed as universally equivalent. We view the settings used here, which were tuned to our mock communities and sequence qualities, as a trade-off between oversplitting some taxa and undersplitting others.

Mock communities provide important tools for optimizing and validating bench and bioinformatic methods. The relatively few fungal ITS mock community studies have typically found weak relationships between starting biomass or template DNA concentration and numbers of reads obtained for a particular taxon (12, 28, 31). In contrast, we observed a strong correlation between the amount of genomic template DNA added to the mixture and the number of reads assigned to an OTU. This result may be due to a lower bias with the new primers and/or our bench methods. In addition, weaker correlations might have been found had we utilized more species. Despite the strong correlations, we still obtained up to 6.7 times more reads from a particular fungus than expected. One of many possible explanations is that fast-growing fungi (such as Spizellomyces and Coprinopsis) may tend to have higher rRNA copy numbers than slow-growing taxa (such as Amanita and Tricholoma), as has been shown in prokaryotes (32, 72). Additional information on copy number variation across the fungal Tree of Life might prove helpful in refining abundance estimates from amplicon sequencing data sets, as it has for prokaryotes (73, 74). Despite the correspondence between expected and observed abundances, we recovered many more OTUs than expected, in part due to apparent contaminant fungi. Contaminants have been reported from nearly all mock community studies to date. In our case, the contaminants occurred at extremely low abundances relative to the intended mock community members. Overall, our results paint a more optimistic picture than several previous studies with respect to accurately capturing fungal taxon abundance by high-throughput amplicon sequencing.

Our results are also encouraging with respect to artifacts and reproducibility. We encountered a very low rate of putative chimeric sequences (0.021%). There are at least two possible explanations. First, the target amplicon is composed mostly of the highly variable ITS2 region, which should be less prone to cross-taxon hybridization and amplification than conserved regions, such as the 5.8S. Low rates of chimera formation were also reported by Ihrmark et al. in their ITS2-targeted 454 study (27). Longer amplicons that span conserved regions appear to be more prone to chimera formation (4, 75). A second possibility is that chimeras are simply more difficult to detect with the relatively short Illumina reads utilized in this study (59).

Using completely different primers and Sanger sequencing of clone libraries versus Illumina amplicon sequencing, we recovered highly congruent portraits of fungal community structure from the same soil samples. In particular, the high Mantel correlation between the two Bray-Curtis distance matrices was remarkable. The similarity in dominant taxa also supports the congruence of the results.

Due to their wide coverage across Fungi, selectivity against nonfungal eukaryotic lineages, and low apparent taxonomic bias, we hope that these new ITS primers and Illumina methods may prove useful in fungal ecology. As read lengths from high-throughput sequencing continue to increase, it may be that longer amplicons that provide more phylogenetic signal can replace ITS1- or ITS2-targeted surveys. However, given the considerable variation in length across the entire ITS1-5.8S-ITS2 region in fungi, if longer amplicons are utilized, it will be important to evaluate and seek to minimize size biases arising from amplification or sequencing steps (27). Our results are also encouraging with respect to prospects for estimating taxon abundances from fungal Illumina amplicon data. Without reliable abundance data, understanding of the drivers of fungal community composition and function will remain limited. To this end, it would be valuable to obtain more information on ribosomal copy number variation and to construct and make available well-characterized phylogenetically diverse mock communities that can be utilized to further optimize and validate fungal amplicon sequencing methods.

## Supplementary Material

Supplemental material
