# Supplementary material for: Accurate Estimation of Fungal Diversity and Abundance through Improved Lineage-Specific Primers Optimized for Illumina Amplicon Sequencing
Source: Appl Environ Microbiol. 2016 Nov 21;82(24):7217–26. doi: 10.1128/AEM.02576-16 (PMC5118932; doi:10.1128/AEM.02576-16)
Supplement: Supplemental material [file supp_82_24_7217__index.html]

Supplemental material 

# Accurate Estimation of Fungal Diversity and Abundance through Improved Lineage-Specific Primers Optimized for Illumina Amplicon Sequencing

## Supplemental material

**Files in this Data Supplement:**

- Supplemental file 1 -

  Observed versus expected read counts for mock communities A and B (Table S1), scripts and analysis settings (Table S2), taxonomic affiliations of intron-containing OTUs (Table S3), full list of mock community OTUs with abundances and identifications (Table S4), affiliations of reads by ITSx (Table S5), alignment of 3? SSU region showing exemplar soil OTUs with and without putative type I self-splicing introns (Fig. S1), alignment of 3? SSU region including an array of fungal and nonfungal taxa and the primers ITS1-F and ITS1 (Fig. S2), predicted order-level coverage for 5.8S-Fun and fITS9 (Fig. S3), and Cluster dendrograms based on fungal community composition derived from Sanger sequencing of clone libraries versus Illumina MiSeq amplicon sequencing (Fig. S4).

  PDF, 2.6M
- Supplemental file 2 -

  Full species and site matrix for soil samples (Data Set S1).

  XLSX, 238K
- Supplemental file 3 -

  Full list of *in silico* validated oligonucleotides (Data Set S2).

  XLSX, 13K
