# Supplementary material for: Accurate Estimation of Fungal Diversity and Abundance through Improved Lineage-Specific Primers Optimized for Illumina Amplicon Sequencing
Source: Appl Environ Microbiol. 2016 Nov 21;82(24):7217–26. doi: 10.1128/AEM.02576-16 (PMC5118932; doi:10.1128/AEM.02576-16)
Supplement: Supplemental material [file AEM.02576-16_zam999117573s1.pdf]

TABLE S1 Observed versus expected read counts for mock communities A and B

		Mock A		Mock B	
**Species**	**Phylum**	**Expected Reads**	**Observed Reads**	**Expected Reads**	**Observed Reads**
Amphinema byssoides	Basidiomycota	33130	28969	8675	6681
Schizosaccharomyces pombe	Ascomycota	32802	25757	859	1699
Tylospora asterophora	Basidiomycota	3280	5642	85892	75171
Mortierella alpina	Mortierellamycotina	3280	5148	86	154
Coprinopsis cinerea	Basidiomycota	3280	10351	859	5817
Tricholoma vacinnium	Basidiomycota	328	111	8589	4926
Spizellomyces punctatus	Chytridiomycota	33	173	8589	37723
Amanita muscaria	Basidiomycota	33	15	85892	67271

TABLE S2 Scripts and analysis settings.

split_libraries_fastq.py
-i INPUT-FILE --phred_offset 33 --barcode_type not-barcoded -q 19 -p 0.70 -r 2 --rev_comp --store_qual_scores --sample_ids SAMPLE -o OUTPUT-FILE
ITSx
-i INPUT-FILE -o OUTPUT-FILE --cpu 8 --preserve T --partial 80 --truncate F -t All --complement F --graphical F
identify_chimeric_seqs.py
-i INPUT-FILE -o OUTPUT-DIRECTORY -m usearch61 --suppress_usearch61_ref
pick_open_reference_otus.py
-i INPUT-FILE -m usearch61 -r sh_refs_qiime_ver7_97_01.08.2015.fasta -a -s 0.1 --suppress_taxonomy_assignment --suppress_align_and_tree -o OUTPUT-DIRECTORY-p PARAMETERS-FILE
assign_taxonomy.py
-i INPUT-FILE -r sh_refs_qiime_ver7_97_01.08.2015.fasta -t sh_taxonomy_qiime_ver7_97_01.08.2015.txt -m blast -o OUTPUT-DIRECTORY

TABLE S3 Taxonomic affiliations of intron-containing OTUs

Intron-containing OTU	Best Match GenBank Accession	Best Match Description	Phylum or Subphylum	Order
OTU691_ITS_091P_05_0119_O_TKN14_3316E7	DQ525501	Pleopsidium chlorophanum Reeb VR8-VIII-02/4	Ascomycota	Acarosporales
OTU817_ITS_106P_04_0119_O_TKN10_3230F20	DQ525501	Pleopsidium chlorophanum Reeb VR8-VIII-02/4	Ascomycota	Acarosporales
OTU639_123P_04_0051_M_TKN10_3232C15	AF274077	Anzina carneonivea	Ascomycota	Agyriales
OTU210_091P_05_0119_O_3310_K10	AF243397	Botryosphaeria corticis 96-125	Ascomycota	Botryosphaeriales
OTU211_127P_04_0051_O_3241I8	AF243397	Botryosphaeria corticis 96-125	Ascomycota	Botryosphaeriales
SEOTU808_068P_04_0119_O_TKN10_3242L10	AF243397	Botryosphaeria corticis 96-125	Ascomycota	Botryosphaeriales
OTU873a_087P_05_0119_O_3318_G14	AY615188	Fusicoccum sp. CMW7022	Ascomycota	Botryosphaeriales
SEOTU1185_ITS_087P_05_0109_O_TKN12_3249N10	AJ972848	Capnobotryella sp. MA 4917	Ascomycota	Capnodiales
OTU121_087P_04_0109_O_TKN8_3208G1	AJ244239	Capnodium coffeae CBS 147.52	Ascomycota	Capnodiales
OTU930_ITS_127P_05_0040_O_TKN14_3315B5	FJ755824	Mycosphaerella sp. F29	Ascomycota	Capnodiales
OTU128_ITS_023P_04_0001_O_TKN7_3183E23	EU019302	Teratosphaeria mexicana CPC 12349	Ascomycota	Capnodiales
SEOTU803_106P_04_0119_O_TKN10_3232B9	AF284128	Capronia sp. UBCTRA 1322.11	Ascomycota	Chaetothyriales
OTU635_106P_05_0040_O_TKN14_3314K18	AF284126	Capronia sp. UBCTRA1522.6	Ascomycota	Chaetothyriales
OTU95_102P_05_0051_O_TKN14_3310M16	AF284126	Capronia sp. UBCTRA1522.6	Ascomycota	Chaetothyriales
OTU96_127P_04_0015_O_TKN8_3200J16	AF050261	Capronia villosa	Ascomycota	Chaetothyriales
OTU636_ITS_123P_04_0126_O_TKN9_3327A19	EU035408	Cladophialophora humicolae CBS 117536	Ascomycota	Chaetothyriales
OTU689_ITS_068P_05_0109_M_TKN12_3250E6	EU035408	Cladophialophora humicolae CBS 117536	Ascomycota	Chaetothyriales
OTU39_ITS_123P_05_0039_O_TKN13_3309L17	EU035408	Cladophialophora humicolae CBS 117536	Ascomycota	Chaetothyriales
OTU506_ITS_038P_05_0126_O_TKN13_3306H14	EU035408	Cladophialophora humicolae CBS 117536	Ascomycota	Chaetothyriales
SEOTU1402_ITS_067P_05_0039_M_TKN13_3322N14	EF016386	Cladophialophora minutissima UAMH 10710	Ascomycota	Chaetothyriales
OTU685_ITS_023P_05_0040_M_TKN14_3315O22	EF016385	Cladophialophora minutissima UAMH 10711	Ascomycota	Chaetothyriales
OTU18_059P_04_0001_M_TKN7_3182A7	AF486119	Phialophora finlandia CBS 444.86	Ascomycota	Chaetothyriales
OTU946_ITS_067P_05_0039_M_TKN13_3309B5	AF486119	Phialophora finlandia CBS 444.86	Ascomycota	Chaetothyriales
OTU20_ITS_087P_04_0001_M_TKN7_3180I24	AF486119	Phialophora finlandia CBS 444.86	Ascomycota	Chaetothyriales
OTU380_123P_05_0022_M_TKN12_3249G2	U66727	Phialophora gregata	Ascomycota	Chaetothyriales
OTU385_ITS_127P_04_0015_O_TKN8_3200P19	DQ069046	Phialophora sp. aurim712	Ascomycota	Chaetothyriales
OTU23_ITS_123P_04_0126_O_TKN9_3243N17	DQ069046	Phialophora sp. aurim712	Ascomycota	Chaetothyriales
SEOTU1341_ITS_106P_05_0040_O_TKN14_3312E7	EU314707	Phialophora sp. DF33	Ascomycota	Chaetothyriales
SEOTU779_ITS_123P_04_0051_M_TKN10_3241J17	AF083200	Phialophora sp. p3847	Ascomycota	Chaetothyriales
OTU138_059P_04_0126_M_TKN9_3329J5	AF083199	Phialophora sp. p3901	Ascomycota	Chaetothyriales
SEOTU599_091P_04_0126_M_TKN9_3328G11	AJ888459	Sarcinomyces sp. MA4760	Ascomycota	Chaetothyriales
OTU504_ITS_087P_05_0119_O_TKN14_3321N23	DQ093680	Lecythophora mutabilis aurim1180	Ascomycota	Coniochaetales
OTU801_ITS_059P_04_0109_O_TKN8_3207I14	AM087275	Cenococcum geophilum 10-5	Ascomycota	Dothideomycetes

OTU520_ITS_102P_04_0039_O_TKN9_3238B21	DQ179119	Cenococcum geophilum Ve-95-12	Ascomycota	Dothideomycetes
OTU680_ITS_087P_05_0126_M_TKN13_3323N8	DQ179119	Cenococcum geophilum Ve-95-12	Ascomycota	Dothideomycetes
OTU952_ITS_059P_05_0126_O_TKN13_3300L18	GU550109	Cenococcum geophilum Hy4	Ascomycota	Dothideomycetes
OTU869_ITS_068P_05_0109_M_TKN12_3249D15	GU092938	Hamigera fusca NRRL 35601	Ascomycota	Eurotiales
OTU522_ITS_038P_05_0126_O_TKN13_3301D12	GU092938	Hamigera fusca NRRL 35601	Ascomycota	Eurotiales
OTU523_ITS_068P_05_0109_M_TKN12_3252K1	GU092938	Hamigera fusca NRRL 35601	Ascomycota	Eurotiales
OTU646_087P_05_0001_M_TKN11_3265D2	AJ133432	Calyptrozyma arxii CBS 354.92	Ascomycota	Eurotiomycetes incertae sedis
OTU686_ITS_068P_04_0119_O_TKN10_3230N1	EU784257	Geoglossum umbratile RBG Kew K(M)120622	Ascomycota	Geoglossales
OTU9_ITS_127P_05_0015_M_TKN12_3253K5	AY789429	Sarcoleotia globosa	Ascomycota	Geoglossales
OTU682_ITS_023P_04_0039_M_TKN9_3243N18	AY789429	Sarcoleotia globosa	Ascomycota	Geoglossales
OTU681_ITS_127P_05_0039_M_TKN13_3307P17	AY789429	Sarcoleotia globosa MBH52476	Ascomycota	Geoglossales
SEOTU1495_ITS_087P_05_0126_M_TKN13_3308A7	AB041243	Allantophomopsis lycopodina:IFO 32908	Ascomycota	Helotiales
OTU683_ITS_087P_05_0001_M_TKN11_3263L16	AB041243	Allantophomopsis lycopodina:IFO 32908	Ascomycota	Helotiales
OTU71_ITS_102P_05_0022_M_TKN12_3271G22	EU998914	Articulospora tetracladia CCM-F-10806	Ascomycota	Helotiales
OTU390_ITS_102P_04_0015_M_TKN8_3197C17	EU998915	Articulospora tetracladia CCM-F-12499	Ascomycota	Helotiales
OTU660_091P_05_0123_M_TKN13_3305I18	EU998915	Articulospora tetracladia CCM-F-12499	Ascomycota	Helotiales
OTU89_ITS_123P_05_0022_M_TKN12_3256J8	AY789421	Bryoglossum gracile MBH52481	Ascomycota	Helotiales
OTU22_ITS_106P_04_0039_O_TKN9_3329I18	EF093179	Cadophora finlandica	Ascomycota	Helotiales
OTU668_ITS_127P_04_0123_M_TKN9_3244P19	EU557316	Cadophora finlandica Simb6s4d-2	Ascomycota	Helotiales
OTU521_ITS_059P_04_0126_M_TKN9_3243D15	EU557316	Cadophora finlandica Simb6s4d-2	Ascomycota	Helotiales
OTU374_ITS_091P_04_0109_M_TKN8_3203I18	EF093179	Cadophora finlandica CFI-3	Ascomycota	Helotiales
OTU21_ITS_038P_05_0012_M_TKN11_3267E24	EF093179	Cadophora finlandica CFI-3	Ascomycota	Helotiales
OTU769_ITS_127P_05_0039_M_TKN13_3305P19	EF093179	Cadophora finlandica CFI-3	Ascomycota	Helotiales
OTU770_ITS_127P_05_0040_O_TKN14_3313B1	EU770236	Cadophora sp. ICMP 17486	Ascomycota	Helotiales
OTU136_091P_04_0109_M_TKN8_3207E1	AY371513	Cadophora sp. NH1-2	Ascomycota	Helotiales
OTU132_102P_05_0012_O_TKN11_3262P16	AY371513	Cadophora sp. NH1-2	Ascomycota	Helotiales
SEOTU1496_068P_05_0123_M_TKN13_3306K21	AY371513	Cadophora sp. NH1-2	Ascomycota	Helotiales
SEOTU611_127P_04_0123_M_TKN9_3324E4	AY371513	Cadophora sp. NH1-2	Ascomycota	Helotiales
SEOTU176_ITS_127P_04_0015_O_TKN8_3201E5	AY112936	cf. Hymenoscyphus sp. he7-23-5	Ascomycota	Helotiales
OTU486_ITS_106P_04_0119_O_TKN10_3235B18	AY112936	cf. Hymenoscyphus sp. he7-23-5	Ascomycota	Helotiales
OTU766_ITS_102P_05_0022_M_TKN12_3272J5	DQ093752	Chalara microchona SM19A2	Ascomycota	Helotiales
OTU494_ITS_102P_05_0022_M_TKN12_3272B13	DQ202512	Cudoniella acicularis CBS 100273	Ascomycota	Helotiales
SEOTU1099_127P_05_0015_M_TKN12_3254A4	DQ195779	Fulvoflamma eucalypti CPC 11243	Ascomycota	Helotiales
OTU36_ITS_127P_05_0039_TKN13_3304J14	EU854569	Geomyces destructans 20682-10	Ascomycota	Helotiales
OTU669_ITS_023P_04_0039_M_TKN9_3244P8	EU854569	Geomyces destructans 20682-10	Ascomycota	Helotiales
OTU853_ITS_023P_05_0001_M_TKN11_3262F5	EU854569	Geomyces destructans 20682-10	Ascomycota	Helotiales

OTU131_067P_04_0012_M_TKN7_3182A22	DQ257357	Holwaya mucida	Ascomycota	Helotiales
OTU16_ITS_091P_04_0001_O_TKN7_3178J18	EU940230	Hyaloscypha fuckelii M233	Ascomycota	Helotiales
OTU26_127P_04_0122_O_3182H2	U59145	Lachnellula calyciformis	Ascomycota	Helotiales
OTU831_ITS_087P_05_0001_M_TKN11_3265K21	U59145	Lachnellula calyciformis	Ascomycota	Helotiales
OTU675_ITS_091P_04_0126_M_TKN9_3238F6	U59145	Lachnellula calyciformis	Ascomycota	Helotiales
OTU947_023P_05_0123_O_TKN13_3323C16	FJ845448	Lachnellula hyalina CBS:185.66	Ascomycota	Helotiales
SEOTU629_ITS_R_TKN9_3327B13	AB481247	Lachnellula subtilissima FC-2354	Ascomycota	Helotiales
OTU663_ITS_091P_04_0126_M_TKN9_3324E12	AF505516	Lachnum hyalopus	Ascomycota	Helotiales
SEOTU895_ITS_002P_05_0122_O_TKN11_3261M23	AF505515	Lachnum lushanense	Ascomycota	Helotiales
OTU673_ITS_023P_04_0039_M_TKN9_3243K16	AM262432	Lachnum pygmaeum 1754	Ascomycota	Helotiales
OTU761_123P_05_0022_M_TKN12_3272M1	AJ430219	Lachnum pygmaeum ARON3255.H	Ascomycota	Helotiales
OTU492_ITS_123P_04_0015_M_TKN8_3200L22	AF505520	Lachnum sclerotii	Ascomycota	Helotiales
OTU674_ITS_091P_05_0119_O_TKN14_3320_E14	AF505520	Lachnum sclerotii	Ascomycota	Helotiales
SEOTU1394_ITS_059P_05_0126_O_TKN13_3303H20	AF505520	Lachnum sclerotii	Ascomycota	Helotiales
SEOTU777_ITS_127P_04_0051_O_TKN10_3233E23	AF505520	Lachnum sclerotii	Ascomycota	Helotiales
OTU496_ITS_023P_04_0039_M_TKN9_3243J5	EU887662	Lachnum sp. 252	Ascomycota	Helotiales
OTU28_ITS_106P_04_0119_O_TKN10_3233B21	AB481269	Lachnum virgineum FC-2143	Ascomycota	Helotiales
OTU906_ITS_087P_05_0119_O_TKN14_3321O22	FJ903334	Leptodontidium elatius E12	Ascomycota	Helotiales
OTU46_127P_04_0122_O_TKN7_3182B4	AY394885	Meliniomyces bicolor	Ascomycota	Helotiales
OTU17_067P_04_0012_M_TKN7_3182H14	AY394885	Meliniomyces bicolor pkc34	Ascomycota	Helotiales
SEOTU216_ITS_127P_04_0015_O_TKN8_3204C11	DQ491498	Mollisia cinerea AFTOL-ID 76	Ascomycota	Helotiales
OTU677_ITS_023P_04_0039_M_TKN9_3325F6	DQ491498	Mollisia cinerea AFTOL-ID 76	Ascomycota	Helotiales
OTU771_ITS_068P_04_0119_O_TKN10_3236P11	DQ491498	Mollisia cinerea AFTOL-ID 76	Ascomycota	Helotiales
SEOTU1265_059P_05_0051_O_TKN14_3318F14	AF141161	Neofabraea malicorticis CBS141.22	Ascomycota	Helotiales
OTU34_023P_04_0001_O_TKN7_3182B9	AY078133	Phialocephala europaea	Ascomycota	Helotiales
OTU881_106P_05_0022_O_TKN12_3258K8	FJ665282	Phialocephala fortinii YWL-78-232a	Ascomycota	Helotiales
OTU501_127P_05_0039_M_TKN13_3323D1	EU434851	Phialocephala sp. 8 UAMH_10206	Ascomycota	Helotiales
OTU33_ITS_038P_04_0022_O_TKN8_3197N10	AY524844	Phialocephala sphaeroides	Ascomycota	Helotiales
OTU32_ITS_102P_05_0012_O_TKN11_3261I8	AY524844	Phialocephala sphaeroides	Ascomycota	Helotiales
OTU498_087P_05_0126_M_TKN13_3301A24	AY524844	Phialocephala sphaeroides UAMH 10279	Ascomycota	Helotiales
SEOTU220_ITS_123P_04_0015_M_TKN8_3202P1	AY524844	Phialocephala sphaeroides UAMH 10279	Ascomycota	Helotiales
OTU484_ITS_106P_04_0022_O_TKN8_3205L24	EU221877	Rhizoscyphus ericae	Ascomycota	Helotiales
OTU15_ITS_067P_04_0012_M_TKN7_3181L10	AY394907	Rhizoscyphus ericae	Ascomycota	Helotiales
OTU14_068P_05_0012_M_TKN11_3261I23	AY394907	Rhizoscyphus ericae pkc29	Ascomycota	Helotiales
OTU376_ITS_087P_04_0109_O_TKN8_3198I4	AY394907	Rhizoscyphus ericae pkc29	Ascomycota	Helotiales
OTU485_ITS_127P_04_0015_O_TKN8_3197F7	AY394907	Rhizoscyphus ericae pkc29	Ascomycota	Helotiales
SEOTU1403_ITS_087P_05_0126_M_TKN13_3302P3	DQ195784	Satchmopsis brasiliensis CBS 420.93	Ascomycota	Helotiales
OTU497_ITS_002P_05_0015_O_TKN12_3256M21	AY187070	Sclerotinia trifoliorum JRS260	Ascomycota	Helotiales

SEOTU894_123P_05_0001_O_TKN11_3263N15	AF250791	Buellia schaereri	Ascomycota	Lecanorales
SEOTU996_ITS_91P_05_0122_O_TKN11_3266H18	DQ534464	Cladonia scabriuscula	Ascomycota	Lecanorales
SEOTU607_ITS_023P_04_0039_M_TKN9_3329E8	EU034666	Hypogymnia physodes Hur H060125	Ascomycota	Lecanorales
OTU417_ITS_123P_04_0015_M_TKN8_3198A5	AF517893	Lepraria neglecta	Ascomycota	Lecanorales
OTU688_ITS_059P_05_0109_O_TKN12_3292E6	AY756474	Micarea deminuta	Ascomycota	Lecanorales
SEOTU1098_068P_05_0109_M_TKN12_3293M5	AY756477	Micarea flagellispora	Ascomycota	Lecanorales
SEOTU1284_ITS_R_TK14_3315_P23	HQ671309	Parmelia sp. Hur R0090039	Ascomycota	Lecanorales
OTU139_102P_04_0122_M_TKN7_3185K20	AY204587	Alatospora acuminata ccm-F 02383	Ascomycota	mitosporic Ascomycota
OTU135_002P_05_0122_O_TKN11_3270A13	AY204589	Alatospora acuminata ccm-F13089	Ascomycota	mitosporic Ascomycota
OTU642_059P_05_0001_O_TKN11_3264E3	AY204589	Alatospora acuminata ccm-F13089	Ascomycota	mitosporic Ascomycota
OTU134_087P_04_0119_M_TKN10_3231G13	AY204589	Alatospora acuminata ccm-F13089	Ascomycota	mitosporic Ascomycota
OTU678_ITS_059P_04_0051_M_TKN10_3237M1	FJ000401	Angulospora sp. NC-2008b	Ascomycota	mitosporic Ascomycota
OTU133_067P_04_0012_M_TKN7_3178F2	AY729939	Flagellospora curvula	Ascomycota	mitosporic Ascomycota
OTU30_ITS_038P_04_0039_M_TKN9_3243C4	DQ093677	Gyoerffyella sp. aurim1226	Ascomycota	mitosporic Ascomycota
OTU882_ITS_127P_05_0039_M_TKN13_3305_L22	EF029201	Spirosphaera beverwijkiana	Ascomycota	mitosporic Ascomycota
OTU661_ITS_127P_05_0015_M_TKN12_3255J22	EF029232	Spirosphaera carici-graminis ICMP15524	Ascomycota	mitosporic Ascomycota
OTU662_ITS_038P_04_0039_M_TKN9_3327E13	EF029232	Spirosphaera carici-graminis ICMP15524	Ascomycota	mitosporic Ascomycota
SEOTU191_ITS_123P_04_0015_M_TKN8_3203N14	DQ248313	Symbiotaphrina buchneri JCM9740	Ascomycota	mitosporic Ascomycota
OTU627_087P_05_0119_O_TKN14_3314O17	DQ351724	Troposporella fumosa	Ascomycota	mitosporic Ascomycota
OTU671_ITS_023P_04_0039_M_TKN9_3326K16	AY706329	Leohumicola minima	Ascomycota	mitosporic Leotiomycetes
SEOTU1338_ITS_023P_05_0040_M_TKN14_3314N1	AY706329	Leohumicola minima DAOM 232587	Ascomycota	mitosporic Leotiomycetes
OTU38_ITS_091P_05_0122_O_TKN11_3266P2	AJ784399	Oidiodendron setiferum	Ascomycota	Myxotrichaceae
OTU124_023P_04_0001_O_TKN7_3178D4	FJ904682	Cryptodiscus pini EB82	Ascomycota	Ostropales
OTU413_106P_04_0022_O_TKN8_3201B14	FJ904682	Cryptodiscus pini EB82	Ascomycota	Ostropales
OTU137_127P_05_0122_M_TKN11_3268N9	AY183370	Pezizomycotina sp. MIRM23	Ascomycota	Pezizales
OTU763_ITS_068P_04_0119_O_TKN10_3231K1	EF596821	Phialea strobilina CBS 643.85	Ascomycota	Pezizales
OTU816_068P_04_0119_O_TKN10_3241C20	AF301420	Terfezia pfeilii Tpa	Ascomycota	Pezizales
OTU887_ITS_068P_05_0109_M_TKN12_3256D10	JX421731	Massarina corticola ASR_H45_31A	Ascomycota	Pleosporales
OTU908_059P_04_0051_M_TKN10_3234J10	DQ529303	Pseudeurotium bakeri MCJAxI	Ascomycota	Pseudeurotiaceae
OTU643_106P_04_0119_O_TKN10_3240P20	DQ529304	Pseudeurotium bakeri MCJAxII	Ascomycota	Pseudeurotiaceae
OTU387_067P_04_0015_O_TKN8_3207B14	EF191244	Coccomyces sp. PRJ-2006b SA22	Ascomycota	Rhytismatales
OTU76_087P_05_0109_O_TKN12_3253C2	AJ293879	Colpoma quercinum C	Ascomycota	Rhytismatales
OTU77_091P_04_0001_O_TKN7_3178L3	AY971733	Lophodermium piceae Lope10	Ascomycota	Rhytismatales
OTU386_ITS_123P_05_0001_O_TKN11_3270I10	AY971733	Lophodermium piceae Lope10	Ascomycota	Rhytismatales
OTU645_087P_05_0126_M_TKN13_3305J2	AF203470	Meloderma desmazieresii	Ascomycota	Rhytismatales
OTU212_023P_05_0123_O_TKN13_3305J10	AY081151	Caloplaca aurantia	Ascomycota	Teloschistales
OTU42_ITS_023P_05_0015_O_TKN12_3256M6	AF096213	Umbilicaria antarctica	Ascomycota	Umbilicariales

OTU29_ITS_091P_05_0109_M_TKN12_3271F12	AF297669	Umbilicaria lyngei	Ascomycota	Umbilicariales
OTU905_ITS_091P_04_0001_O_TKN7_3184K13	FJ475724	Uncultured Sordariomycetes AhedenF16	Ascomycota	Unknown
OTU88_002P_04_0123_O_TKN9_3324C15	FJ475710	Uncultured Verrucariales AhedenH4	Ascomycota	Verrucariales
OTU883_ITS_002P_05_0015_O_TKN12_3271B22	DQ974769	Sebacina sp. src725	Basidiomycota	Sebacinales
OTU90_068P_05_0012_M_TKN11_3261P23	AM113719	Environmental fungal clone MH1-2	Unknown	

TABLE S4 Full list of mock community OTUs with abundances and identifications.

Mock A

OTU	Abundance	Taxonomy	E value	UNITE SH
Dan12_Amphinema_byssoides	28969	k__Fungi;p__Basidiomycota;c__Agaricomycetes;o__Atheliales;f__Atheliaceae;g__Amphinema;s__Amphinema byssoides	6.00E-95	SH015694.07FU_UDB0082
Broad124_Schizosaccharomyces_pombe	25757	No blast hit	None	None
KT804081_Coprinopsis_cinerea_OT2_180	10351	k__Fungi;p__Basidiomycota;c__Agaricomycetes;o__Agaricales;f__Psathyrellaceae;g__Coprinopsis;s__Coprinopsis calospora	1.00E-68	SH030798.07FU_JX118675
Dan6_Tylospora_asterophora	5642	k__Fungi;p__Basidiomycota;c__Agaricomycetes;o__Atheliales;f__Atheliaceae;g__Tylospora;s__Tylospora sp	7.00E-79	SH015563.07FU_UDB0026
Mortierella_alpina	5148	k__Fungi;p__Zygomycota;c__Incertae sedis;o__Mortierellales;f__Mortierellaceae;g__Mortierella;s__Mortierella amoeboidea	3.00E-116	SH006229.07FU_HQ63034
EU379241_Spizellomyces_punctatus_SW001	173	k__Fungi;p__Chytridiomycota;c__Chytridiomycetes;o__Spizellomycetales;f__Spizellomycetaceae;g__Spizellomyces;s__Spizellomyces punctatus	2.00E-120	SH018989.07FU_AY99709
Dan2_Tricholoma_vaccinum	111	k__Fungi;p__Basidiomycota;c__Agaricomycetes;o__Agaricales;f__Tricholomataceae;g__Tricholoma;s__Tricholoma vaccinum	4.00E-74	SH031061.07FU_UDB0116
Dan9_Amanita_muscaria	15	k__Fungi;p__Basidiomycota;c__Agaricomycetes;o__Agaricales;f__Amanitaceae;g__Amanita;s__Amanita muscaria	5.00E-67	SH003263.07FU_UDB0158
New.CleanUp.ReferenceOTU11	4	k__Fungi;p__Basidiomycota;c__Agaricomycetes;o__Agaricales;f__Psathyrellaceae;g__Coprinopsis;s__Coprinopsis calospora	3.00E-56	SH030798.07FU_JX118675
New.CleanUp.ReferenceOTU6	4	k__Fungi;p__Ascomycota;c__Leotiomycetes;o__Helotiales;f__Incertae sedis;g__Cadophora;s__Cadophora finlandica	6.00E-64	SH027361.07FU_AF48611
New.CleanUp.ReferenceOTU41	3	k__Fungi;p__Basidiomycota;c__Agaricomycetes;o__Atheliales;f__Atheliaceae;g__Amphinema;s__Amphinema byssoides	1.00E-43	SH015694.07FU_UDB0082
New.CleanUp.ReferenceOTU5	3	k__Fungi;p__Basidiomycota;c__Agaricomycetes;o__Agaricales;f__Cortinariaceae;g__Cortinarius;s__Cortinarius sp	5.00E-43	SH009343.07FU_KF61765
New.CleanUp.ReferenceOTU9	3	k__Fungi;p__Basidiomycota;c__Agaricomycetes;o__Agaricales;f__Cortinariaceae;g__Cortinarius;s__Cortinarius rigens	1.00E-89	SH009351.07FU_JF907880
New.CleanUp.ReferenceOTU0	2	k__Fungi;p__Basidiomycota;c__Agaricomycetes;o__Agaricales;f__Hygrophoraceae;g__Hygrophorus;s__Hygrophorus korhonenii	7.00E-76	SH026191.07FU_UDB0005
New.CleanUp.ReferenceOTU1	2	k__Fungi;p__Basidiomycota;c__Agaricomycetes;o__Agaricales;f__Inocybaceae;g__Inocybe;s__Inocybe leiocephala	2.00E-85	SH031324.07FU_AM8827
New.CleanUp.ReferenceOTU23	2	k__Fungi;p__Ascomycota;c__Leotiomycetes;o__Helotiales;f__Hyaloscyphaceae;g__Lachnum;s__Lachnum sp	5.00E-46	SH018445.07FU_UDB0204
New.CleanUp.ReferenceOTU26	2	k__Fungi;p__Basidiomycota;c__Agaricomycetes;o__Agaricales;f__Cortinariaceae;g__Cortinarius;s__Cortinarius sp OTU242	2.00E-51	SH009427.07FU_HQ21575
New.CleanUp.ReferenceOTU30	2	k__Fungi;p__Basidiomycota;c__Agaricomycetes;o__Atheliales;f__Atheliaceae;g__Piloderma;s__Piloderma sp	3.00E-81	SH014858.07FU_UDB0017
New.CleanUp.ReferenceOTU31	2	k__Fungi;p__Basidiomycota;c__Agaricomycetes;o__Agaricales;f__Strophariaceae;g__Hebeloma;s__Hebeloma sp ME12_A3	4.00E-77	SH030730.07FU_JX436892
New.CleanUp.ReferenceOTU4	2	k__Fungi;p__Basidiomycota;c__Agaricomycetes;o__Agaricales;f__Psathyrellaceae;g__Coprinopsis;s__Coprinopsis calospora	2.00E-12	SH030798.07FU_JX118675
New.CleanUp.ReferenceOTU54	2	k__Fungi;p__Basidiomycota;c__Agaricomycetes;o__Atheliales;f__Atheliaceae;g__Amphinema;s__Amphinema sp	3.00E-72	SH015695.07FU_UDB0017
Grand Total	76199			
Contaminant Total	22			
Percent Contaminant	0.02887177			
Contaminant OTUs	9			

Mock B

OTU	Abundance	Taxonomy	E value	UNITE SH
Dan6_Tylospora_asterophora	75171	k__Fungi;p__Basidiomycota;c__Agaricomycetes;o__Atheliales;f__Atheliaceae;g__Tylospora;s__Tylospora sp	7.00E-79	SH015563.07FU_UDB0026
Dan9_Amanita_muscaria	67271	k__Fungi;p__Basidiomycota;c__Agaricomycetes;o__Agaricales;f__Amanitaceae;g__Amanita;s__Amanita muscaria	2.00E-70	SH003263.07FU_UDB0158
EU379241_Spizellomyces_punctatus_SW001	37723	k__Fungi;p__Chytridiomycota;c__Chytridiomycetes;o__Spizellomycetales;f__Spizellomycetaceae;g__Spizellomyces;s__Spizellomyces punctatus	2.00E-120	SH018989.07FU_AY99709
Dan12_Amphinema_byssoides	6681	k__Fungi;p__Basidiomycota;c__Agaricomycetes;o__Atheliales;f__Atheliaceae;g__Amphinema;s__Amphinema byssoides	6.00E-95	SH015694.07FU_UDB0082
KT804081_Coprinopsis_cinerea_OT2_180	5817	k__Fungi;p__Basidiomycota;c__Agaricomycetes;o__Agaricales;f__Psathyrellaceae;g__Coprinopsis;s__Coprinopsis calospora	1.00E-68	SH030798.07FU_JX118675
Dan2_Tricholoma_vaccinum	4926	k__Fungi;p__Basidiomycota;c__Agaricomycetes;o__Agaricales;f__Tricholomataceae;g__Tricholoma;s__Tricholoma vaccinum	4.00E-74	SH031061.07FU_UDB0116
Broad124_Schizosaccharomyces_pombe	1699	No blast hit	None	None
Mortierella_alpina	154	k__Fungi;p__Zygomycota;c__Incertae sedis;o__Mortierellales;f__Mortierellaceae;g__Mortierella;s__Mortierella amoeboidea	3.00E-116	SH006229.07FU_HQ63034
New.CleanUp.ReferenceOTU0	81	k__Fungi;p__Ascomycota;c__Saccharomycetes;o__Saccharomycetales;f__Saccharomycetaceae;g__Saccharomyces;s__Saccharomyces cerevisiae	2.00E-72	SH004658.07FU_AB01804
New.CleanUp.ReferenceOTU22	6	k__Fungi;p__Basidiomycota;c__Agaricomycetes;o__Atheliales;f__Atheliaceae;g__Tylospora;s__Tylospora sp	1.00E-52	SH015563.07FU_UDB0026
New.CleanUp.ReferenceOTU2	5	k__Fungi;p__Basidiomycota;c__Agaricomycetes;o__Atheliales;f__Atheliaceae;g__Amphinema;s__Amphinema byssoides	1.00E-68	SH015694.07FU_UDB0082
New.CleanUp.ReferenceOTU1	4	k__Fungi;p__Basidiomycota;c__Agaricomycetes;o__Atheliales;f__Atheliaceae;g__Piloderma;s__Piloderma sp	8.00E-82	SH014858.07FU_UDB0017
New.CleanUp.ReferenceOTU16	4	k__Fungi;p__Basidiomycota;c__Agaricomycetes;o__Agaricales;f__Inocybaceae;g__Inocybe;s__Inocybe giacomi	5.00E-83	SH013420.07FU_AM8827
New.CleanUp.ReferenceOTU11	3	k__Fungi;p__Basidiomycota;c__Agaricomycetes;o__Atheliales;f__Atheliaceae;g__Piloderma;s__Piloderma sp	5.00E-74	SH015684.07FU_JQ39309
New.CleanUp.ReferenceOTU17	2	k__Fungi;p__Basidiomycota;c__Agaricomycetes;o__Atheliales;f__Atheliaceae;g__Amphinema;s__Amphinema byssoides	9.00E-66	SH015694.07FU_UDB0082
New.CleanUp.ReferenceOTU24	2	k__Fungi;p__Basidiomycota;c__Agaricomycetes;o__Russulales;f__Russulaceae;g__Lactarius;s__Lactarius sp	3.00E-59	SH031579.07FU_FJ196934
New.CleanUp.ReferenceOTU31	2	k__Fungi;p__Ascomycota;c__Leotiomycetes;o__unidentified;f__unidentified;g__unidentified;s__Leotiomycetes sp	3.00E-53	SH004600.07FU_GQ99615
New.CleanUp.ReferenceOTU36	2	k__Fungi;p__Basidiomycota;c__Agaricomycetes;o__Atheliales;f__Atheliaceae;g__Amphinema;s__Amphinema sp	2.00E-98	SH015695.07FU_UDB0017
New.CleanUp.ReferenceOTU38	2	k__Fungi;p__Basidiomycota;c__Agaricomycetes;o__Polyporales;f__Phanerochaetaceae;g__Phanerochaete;s__Phanerochaete sp	3.00E-94	SH011584.07FU_UDB0142
New.CleanUp.ReferenceOTU5	2	k__Fungi;p__Basidiomycota;c__Agaricomycetes;o__Agaricales;f__Cortinariaceae;g__Dermocybe;s__Dermocybe alienata	4.00E-53	SH033108.07FU_GU23332
New.CleanUp.ReferenceOTU9	2	k__Fungi;p__Basidiomycota;c__Agaricomycetes;o__Agaricales;f__unidentified;g__unidentified;s__Agaricales sp	4.00E-80	SH017806.07FU_JX436916
TOTAL	199559			
Contaminant Total	102			
Percent Contaminant	0.0511127			
Contaminant OTUs	9			

TABLE S5 Affiliations of reads by ITSx.

Lineage	Mock A	Mock B	T51O4	UP1BM5	UP1BO5	UP3AM5	UP3AO5	TOTAL
Alveolates	0	0	1	0	0	0	1	2
Amoebozoa	0	0	31	1	7	5	4	48
Bacillariophyta	0	0	2	0	0	3	0	5
Brown algae	0	0	0	0	0	0	0	0
Bryophytes	0	0	0	1	0	0	0	1
Euglenozoa	0	0	0	0	0	0	0	0
Eustigmatophytes	0	0	1	4	1	0	0	6
Fungi	66265	193960	282308	248965	179069	215490	213362	1399419
Green algae	0	0	17	29	138	6	12	202
Liverworts	19	14	920	69	416	109	537	2084
Metazoa	9954	5623	30	101	19	79	47	15853
Microsporidia	0	0	0	0	0	0	0	0
Oomycetes	1	0	0	0	3	0	0	4
Prymnesiophytes	0	0	0	0	0	0	0	0
Raphidophytes	0	0	0	0	0	0	0	0
Red algae	3	3	3	2	6	9	2	28
Rhizaria	0	0	0	4	1	20	2	27
Synurophyceae	0	0	0	0	0	3	8	11
Tracheophyta	2	0	14471	3261	4791	3131	5922	31578
TOTALS	76244	199600	297784	252437	184451	218855	219897	1449268
% non-fungal	13.09	2.83	5.20	1.38	2.92	1.54	2.97	3.44

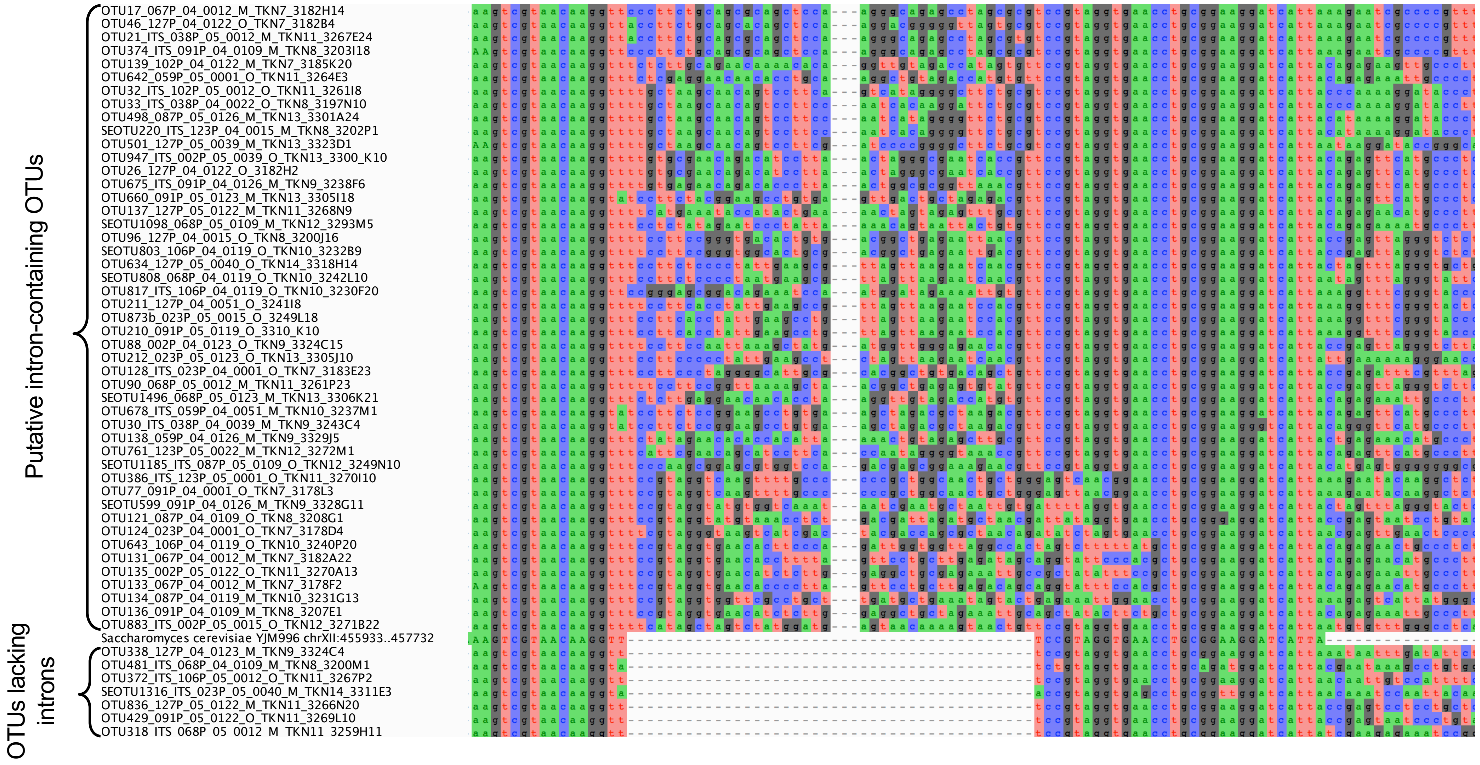

FIG S1 Alignment of 3' SSU region showing exemplar soil OTUs with and without putative Type I self-splicing introns. These OTUs are from the Sanger-sequenced clone library dataset of Taylor et. al., 2014. These cloned fragments were originally amplified using primers ITS1-FL and TW13.

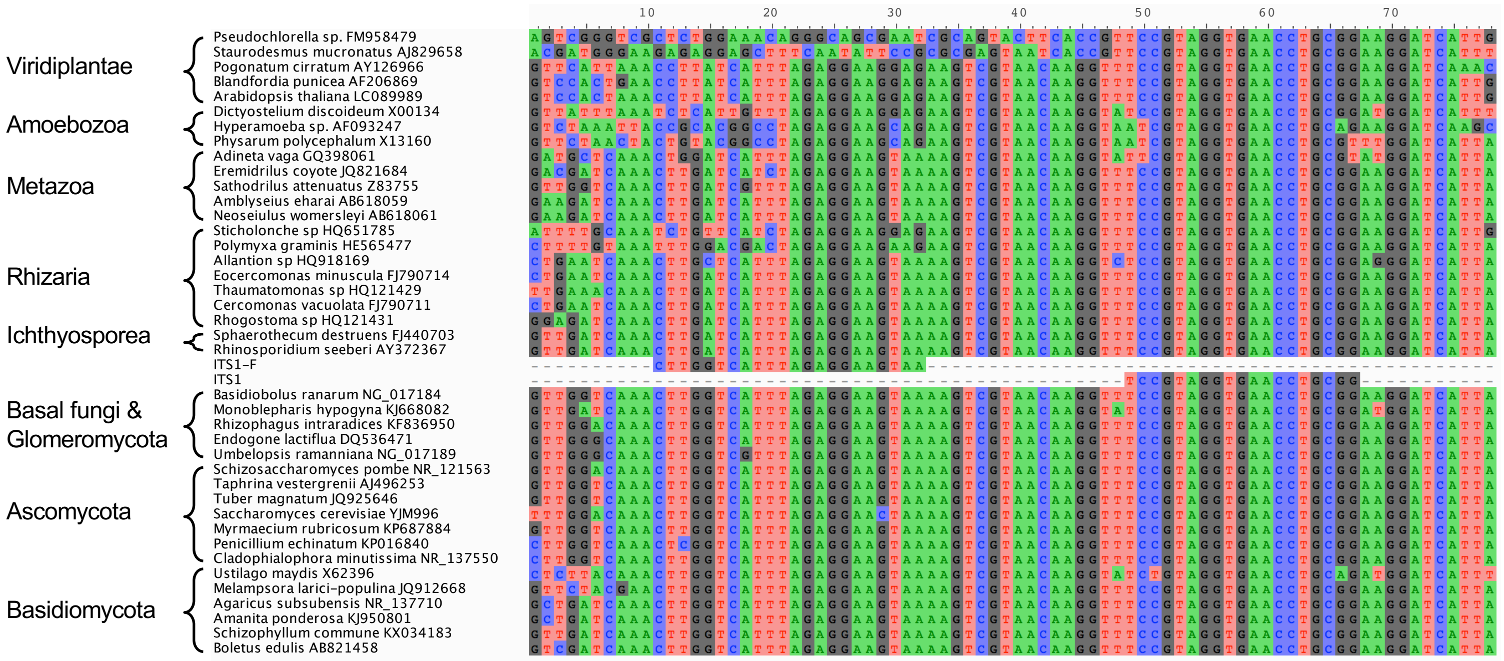

FIG S2 Alignment of 3' SSU region including an array of fungal and non-fungal taxa and the primers ITS1-F and ITS1. Note that ITS1-F has critical mismatches to plant (Viridiplantae) and slime mold (Amoebozoa) sequences, at the 3' end while ITS1 matches most eukaryotes. However, ITS1-F is a moderately good match to various protist lineages, particularly among the Rhizaria and Ichthyosporea.

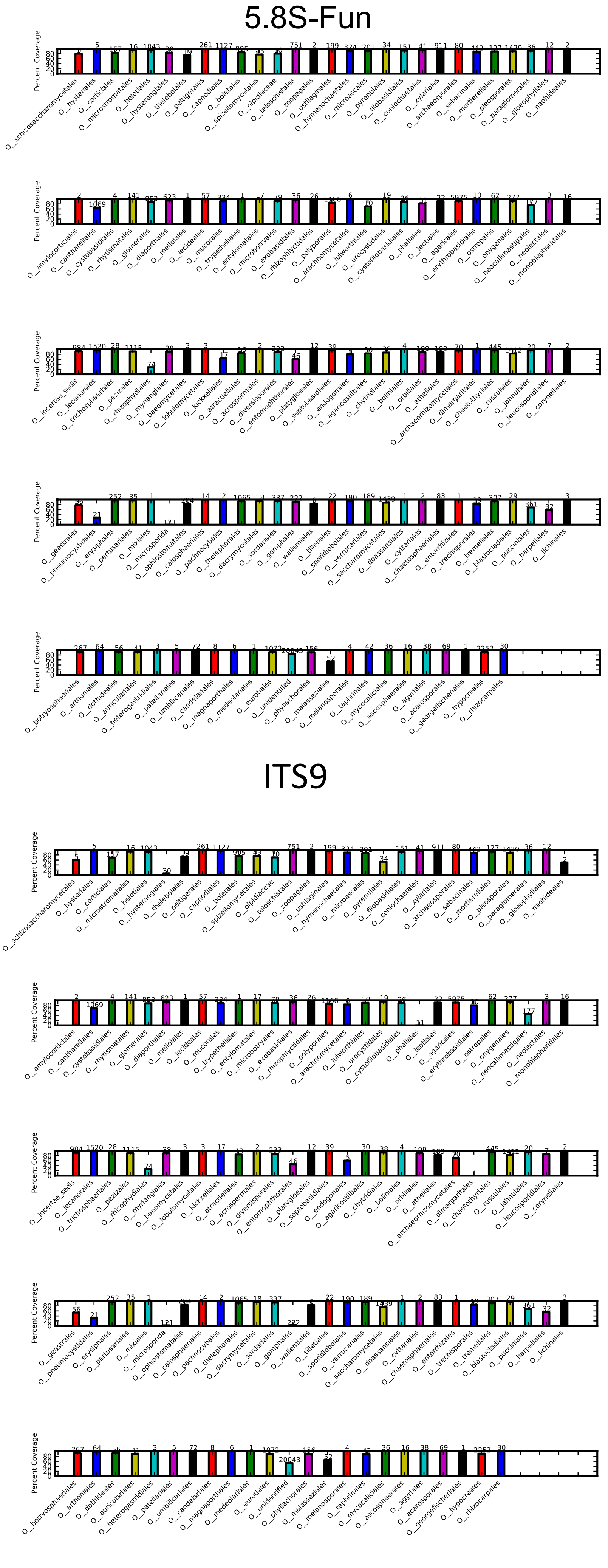

FIG S3 Predicted order-level coverage for 5.8S-Fun and fITS9. PrimerProspector output where the Y axis shows the proportion of taxa in the input alignment that are predicted to amplify with a given primer, the count over each column indicates the number of representative sequences for that group, and the X axis groups taxa into fungal order. We used the entire UNITE 97 database including representatives of all fungal ITS OTUs from GenBank as input sequences for these analyses.

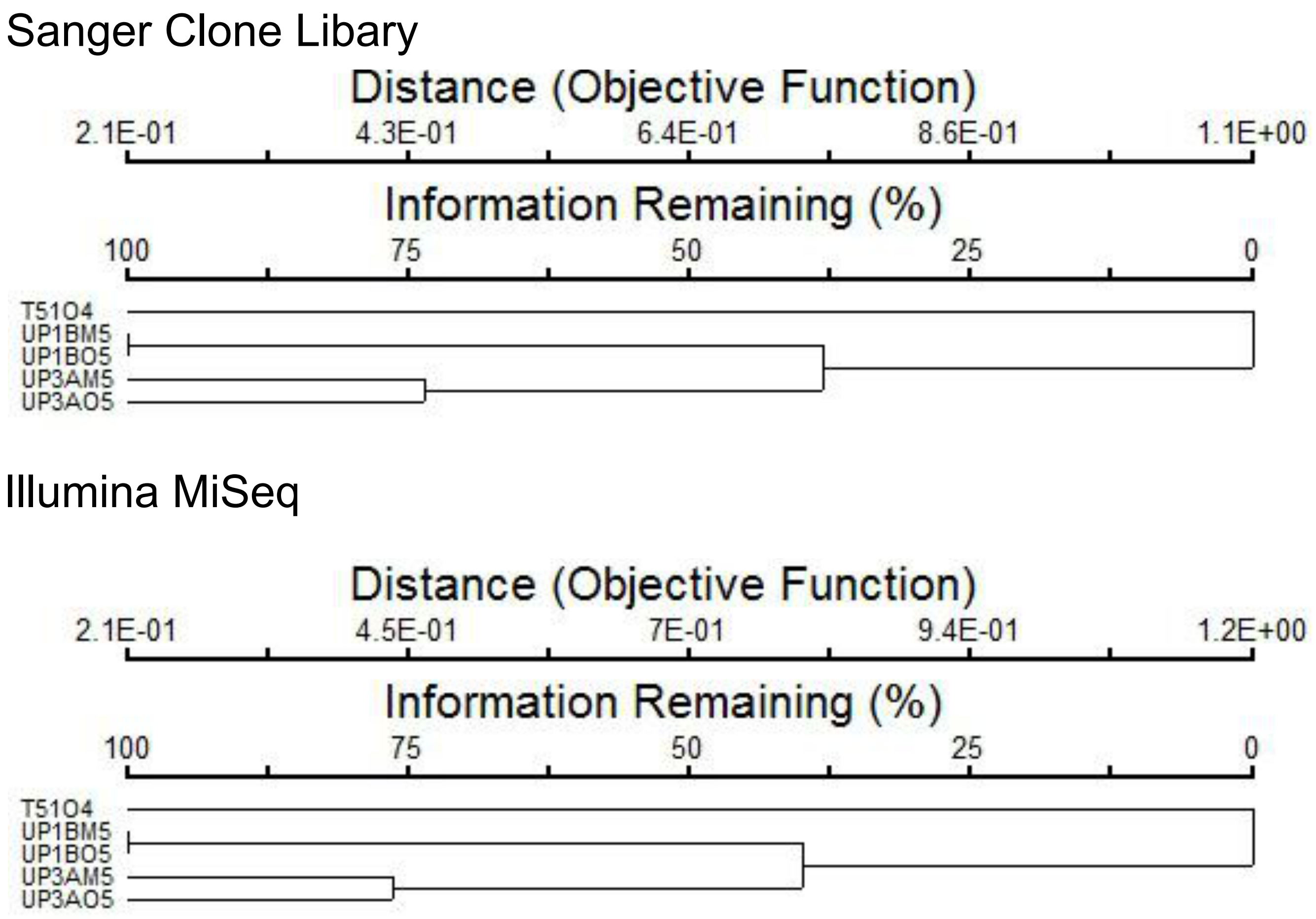

FIG S4 Cluster dendrograms based on fungal community composition derived from Sanger sequencing of clone libraries versus Illumina MiSeq amplicon sequencing. Bray-Curtis abundance-based sample distances were used with the average linkage method. Note that Sanger data are based on a much longer amplicon generated using primers ITS1-F and TW13.
